# Phase Ib and pharmacokinetics study of alpelisib, a PIK3CA inhibitor, and capecitabine in patients with advanced solid tumors

**DOI:** 10.3389/fonc.2024.1390452

**Published:** 2024-07-12

**Authors:** Ah Reum Lim, Boyeon Kim, Jwa Hoon Kim, Myung Han Hyun, Kyong Hwa Park, Yeul Hong Kim, Soohyeon Lee

**Affiliations:** ^1^ Division of Medical Oncology, Department of Internal Medicine, Korea University Anam Hospital, Korea University College of Medicine, Seoul, Republic of Korea; ^2^ Korea University Cancer Research Institute, Korea University College of Medicine, Seoul, Republic of Korea

**Keywords:** metastatic cancer, alpelisib, capecitabine, PIK3CA mutation, advanced solid tumors

## Abstract

**Background:**

This phase Ib study was performed to determine the safety of combination capecitabine with alpleisib (phosphatidylinositol 3-kinase catalytic subunit p110α blockade) and determine the maximal tolerated dose (MTD) and recommended phase ll dose (RP2D) of this combination regimen in patients with advanced solid tumors refractory to standard therapy. The synergistic anti-tumor activity and pharmacokinetics (PK) were investigated.

**Methods:**

Dose escalation phases were conducted in patients with advanced solid cancers who were refractory to standard therapy regardless of *PIK3CA* mutation. Patients were administered orally once daily alpelisib (200mg and 300mg) and twice daily capecitabine (850mg, 1000mg, 1250mg orally, days 1–14) every 3 weeks. Standard “3 + 3” dose escalation was used to define the MTD. The effect of alpelisib on the PK of capecitabine was assessed.

**Results:**

Patients with 6 colorectal cancer (three *PIK3CA* mutation) and 6 breast cancer (all *PIK3CA* mutation) were enrolled. The first three patients in dose level 0 (alpelisib 200mg daily, capecitabine 1,000 mg/m^2^ twice daily) had no dose-limiting toxicities (DLTs). In dose level 1 (alpelisib increased to 300 mg daily, capecitabine 1,000mg twice daily), one of six patients had DLT (grade (Gr) 3 hyperglycemia). When dose level 2 (alpelisib 300mg daily, capecitabine 1,250 mg/m^2^ twice daily) was expanded to 3 patients, no patients had DLTs. The combination of alpelisib 300mg daily and capecitabine 1,250 mg/m^2^ twice daily was declared as the MTD/RP2D in patients with advanced solid tumors. The most common AEs were Gr 1–3 hyperglycemia (75.0%). Frequent all-grade, treatment-related AEs included Gr 2–3 nausea (75.0%), Gr 1–2 diarrhea (50.0%), Gr 1–2 hand-foot syndrome (41.7%), Gr 1–2 anorexia (41.7%), Gr 2 mucositis (33.3%). Antitumor activity was observed in patients with *PIK3CA* mutant breast cancer (3 partial response and 3 stable disease of total 6 patients). Alpelisib exposure (C_max_ and AUC_0-12_) was unaffected by concomitant capecitabine. There were no clinically relevant drug-drug interactions observed between alpelisib and capecitabine.

**Conclusions:**

The combination of alpelisib and capecitabine is generally tolerated, without pharmacokinetic interactions, and shows antitumor activity in patients with *PIK3CA* mutant advanced cancers.

## 1 Introduction

The phosphoinositide 3-kinase (PI3K) pathway is frequently altered in human cancer, reported at a rate of nearly 30% in solid cancer (1–3). Aberrant activation of this pathway is associated with cancer cell proliferation, cell survival, and angiogenesis (4–6). The PIK3CA gene (the phosphatidylinositol-4,5bisphosphate 3-kinase catalytic subunit alpha gene) is the most frequently altered PI3K isoform in solid tumors, with gain-of-function mutations upregulating downstream AKT-mTOR signaling pathways that promote cancer cell growth and proliferation (7, 8). Alpelisib, an oral selective inhibitor of class I PI3K p110α (9, 10), has demonstrated antitumor properties in preclinical studies, particularly in the *PIK3CA*-mutated cancer models (10). In a first-in-human phase I study with advanced solid malignancies with *PIK3CA* alterations, alpelisib showed encouraging preliminary activity and a tolerable safety profile, with the MTD declared as 400 mg once daily (11). However, the clinical efficacy of single-agent alpelisib has been limited, suggesting that combination therapy may be more effective. Alpelisib has been combined with multiple anticancer drugs, such as fulvestrant (12, 13), everolimus (14), olaparib (15), encorafenib (16), paclitaxel (17), cisplatin (18), and cetuximab (19). Based on the results of the SOLAR-1 trial, alpelisib has been approved in combination with the fulvestrant for the treatment of *PIK3CA*-mutated, HR(hormone receptor)-positive, HER2(human epidermal growth factor receptor 2)-negative advanced breast cancer who had received endocrine therapy previously (12). Currently, Alpelisib is being studied in different combinations, expecting to improve synergy effects and overcome resistance to chemotherapy.

Capecitabine is an oral prodrug of 5-FU (fluorouracil) that is effective as chemotherapy treatment for multiple cancers, including colorectal (20, 21), gastric (22), pancreatic (23), and breast cancer (24). As a monotherapy, it has been widely used for salvage regimens in Korea based on retrospective data with a moderate disease control rate of 60% (25). Capecitabine has demonstrated safety and anti-tumor activity when used in combination with other chemotherapy or biological agents such as bevacizumab and lapatinib (26, 27). Additionally, the combination of capecitabine with an mTOR inhibitor targeting the PI3K/Akt/mTOR signaling pathway has shown antitumor effects and tolerable safety (28, 29). Preclinical data has also suggested that combining PI3K inhibition with 5-fluorouracil could increase the anticancer effects in cancer cell lines (30).

In this phase I trial, we evaluated the combination of capecitabine and alpelisib in patients with advanced solid cancer to determine its safety, tolerability, and pharmacokinetic interaction. This phase I trial aimed to assess whether this combination therapy would be a feasible and effective treatment option, especially for patients with *PIK3CA* mutant tumors.

## 2 Material and methods

### 2.1 Study design

This phase Ib, open-label, single-arm study (ClinicalTrials.gov identifier: NCT04753203) enrolled patients with advanced solid tumors using a standard 3 + 3 dose escalation design. The primary objective of a phase Ib was to determine the MTD and RP2D of alpelisib in combination with capecitabine. Secondary objectives included assessment of dose-limiting toxicity (DLT), safety, pharmacokinetics (PK), and preliminary anti-tumor activity of alpelisib plus capecitabine combination. The exploratory objective included biomarker analysis for efficacy and resistance of the alpelisib plus capecitabine combination.

### 2.2 Patients

The dose-escalation phase Ib enrolled patients with a histologically-confirmed, advanced/recurrent solid tumor who have progressed on standard therapy or whose disease does not have established standard therapy and is not limited to *PIK3CA* mutation.

Eligibility criteria included: Eastern Cooperative Oncology Group performance status (ECOG PS) of ≤1, adequate bone marrow and organ function, and life expectancy of ≥3 months. Patients diagnosed with diabetes, impaired glucose tolerance [with a blood glucose of 140–199 mg/dL after a 2-hour oral glucose tolerance test (75g)], previous history of gestational diabetes, or steroid-induced diabetes were not included. The full list of inclusion and exclusion criteria is in **Supplementary Table 1**.

### 2.3 Study treatment

Patients were administered orally once daily alpelisib (200mg and 300mg) and twice daily capecitabine (850mg, 1000mg, 1250mg orally, days 1–14) every 3 weeks. A total of 4 dose levels (**Supplementary Table 2**) was planned, and a standard “3 + 3” design was applied to define the MTD. The MTD was defined as the highest dose at which DLT was experienced by <33% of patients in the first treatment cycle. The RP2D was determined according to the MTD in phase Ib.

DLT was defined as treatment-related adverse events (AEs); DLT did not include adverse events associated with the disease (e.g., symptomatic deterioration due to tumor progression). DLT assessment was only performed during the first cycle and was based on NCI-CTCAE (version 5.0). The evaluation period of DLT was 21 days (i.e., one cycle treatment period), and a subject was considered evaluable for DLT only if capecitabine was administered all 2 weeks and alpelisib was administered 75% or more (That is, the completion of taking at least 16 days out of a total of 21days). DLT included: ≥ grade 2 hyperglycemia (FPG >160 – 250 mg/dL; confirmed with a repeat FPG within 24 hrs.) that did not resolve to grade 1 or less (< 140 mg/dL) within 21 consecutive days (after initiation of oral anti-diabetic treatment); specified grade ≥ 3 hematologic, renal, hepatic, metabolic, or dermatological AEs lasting for 7 days; or any other grade 3 toxicity.

### 2.4 Study assessments

#### 2.4.1 Efficacy

Efficacy parameters were defined using the RECIST (Response Evaluation Criteria in Solid Tumors), version 1.1. The efficacy end-points were progression-free survival (PFS), objective response rate (ORR), duration of response (DOR), disease control rate (DCR), and overall survival (OS).

#### 2.4.2 Safety

Physical examinations including vital signs, body weight, and ECOG performance status were assessed at screening, every 3 weeks. Adverse events and laboratory safety assessment according to NCI CTCAE version 5.0. Safety was monitored throughout the treatment period by the collection of AEs.

#### 2.4.3 Pharmacokinetics

The pharmacokinetic (PK) analysis was conducted in all patients who received at least one dose of alpelisib and capecitabine combination therapy. Blood samples were collected at specified time points, including pre-dose, 1, 2, 4, and 6 hours post-dose, on day 1 of cycle 1. Measurements of alpelisib and capecitabine were carried out with a validated LC-MS/MS assay. PK parameters, including maximum plasma concentration (Cmax), time to reach maximum plasma concentration (tmax), area under the plasma concentration-time curve (AUC), and elimination half-life (t1/2), were calculated using non-compartmental methods. The PK parameters of alpelisib and capecitabine were analyzed separately, and the effect of dose on PK was evaluated. The data were presented as the mean (range) and standard deviation (SD).

#### 2.4.4 *PIK3CA* mutational analysis

*PIK3CA* status was assessed by next-generation sequencing (NGS) using archival or fresh tumor biopsy samples collected before the study started. Specimens were prepared from formalin-fixed and paraffin-embedded tumor tissue. Targeted sequencing was performed using K-MASTER Cancer Panel.

### 2.5 Ethics approval and consent to participate

The clinical trial was approved by the institutional review boards of all participating institutions (2020AN0539) and by the FDA. All procedures involving human participants were carried out in accordance with the Declaration of Helsinki. Written, informed consent was obtained from patients or guardians before enrolment in the study.

## 3 Results

### 3.1 Patient characteristics

Between February 2021, and August 2021, we enrolled 12 patients with advanced solid tumors from Korea University Anam Hospital in Korea and received escalating doses of alpelisib and capecitabine. The first patient registered on 25/02/2021. The median age of enrolled patients was 53.7 years old (range, 33–69) and median number of prior systemic therapies was 3.5 (range, 2–14). 6 patients had colorectal cancer, and 6 patients had breast cancer (including 9 patients with confirmed *PIK3CA* mutation, 3 colorectal cancer, and 6 breast cancer). At the time of data cutoff (April 2023), three patients with breast cancer remained on treatment, with a median follow-up of 3 months (1–26.5 months) (**Table 1**).

**Table 1:** Characteristics of patients.

	Dose level 0 Alpelisib 200mg+ Capeciabine 1000mg/m^2^ (n=3)	Dose level 1 Alpelisib 300mg+ Capeciabine 1000mg/m^2^ (n=6)	Dose level 2 Alpelisib 300mg+ Capeciabine 1250mg/m^2^ (n=3)	Total (n = 12)
Age	50.7 (49–53)	54.5 (33–69)	55.0 (40–63)	53.7 (33–69)
Gender					
Male Female	0 (0) 3 (100)	1 (17) 5 (83)	1 (33) 2 (67)	2 (17) 10 (83)
ECOG PS					
0 1	0 (0) 3 (100)	0 (0) 6 (100)	0 (0) 3 (100)	0 (0) 12 (100)
Diagnosis					
Breast Colorectal	2 (67) 1 (33)	3 (50) 3 (50)	1 (33) 2 (67)	6 (50) 6 (50)
No. of metastatic sites					
1	0 (0)	3 (50)	1 (33)	4 (33)
2	1 (33)	1 (17)	1 (33)	3 (25)
More than 2	2 (67)	2 (33)	1 (33)	5 (42)
Prior anticancer treatment					
Surgery Radiotherapy Chemotherapy	3 (100) 3 (100) 3 (100)	6 (100) 3 (50) 6 (100)	3 (100) 1 (33) 3 (100)	12 (100) 7 (58) 12 (100)
Prior chemotherapy regimens, n					
≤2	2 (67)	2 (33)	3 (100)	7 (58)
3–4	1 (33)	2 (33)	0 (0)	3 (25)
≥5	0	2 (33)	0 (0)	2 (17)

Data are frequency (percentage) or mean (range). ECOG PS, Eastern Cooperative Oncology Group performance status.

### 3.2 Dose escalation and dose-limiting toxicities

The first three patients in dose level 0 (alpelisib 200mg daily, capecitabine 1000 mg/m^2^ twice daily) had no dose-limiting toxicities (DLTs). In dose level 1 (alpelisib increased to 300 mg daily, capecitabine 1000mg twice daily), one of six patients had DLT (grade (Gr) 3 hyperglycemia). When dose level 2 (alpelisib 300mg daily, capecitabine 1250 mg/m^2^ twice daily) was expanded to 3 patients, no patients had DLTs. The combination of alpelisib 300mg daily and capecitabine 1250 mg/m^2^ twice daily was declared the MTD/RP2D in patients with advanced solid tumors.

### 3.3 Efficacy

**Figure 1:**
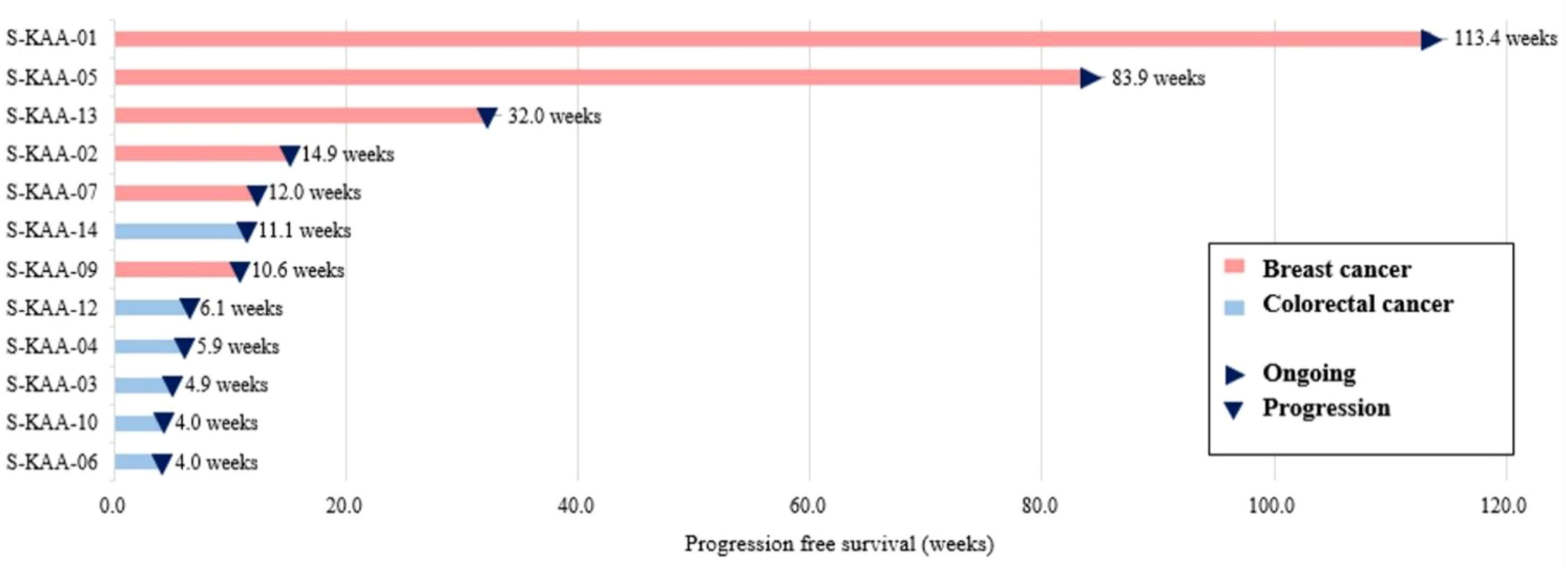
Swimmer’s plot of patients undergoing capecitabine and alpelisib (n=12). Each individual progression free survival (PFS) is plotted from time of first dose (x-axis). Arrows indicate that no disease progression was found during the study period.

The overall objective response rate (ORR) was 25.0% (3 of 12 patients), and the disease control rate (DCR) was 50.0% (6 of 12 patients). Three patients had a partial response, and three had stable disease. The median duration of response in patients with a partial response was 12.5 months (range, 3.5–26.5). The median PFS was 5.9 months (range 0.9–26.5), and the median OS was 9.7 months. At the time of the data cutoff, nine patients died, and three patients survived. The duration of treatment varied depending on the type of cancer. Colorectal cancer patients received almost two cycles of treatment, but breast cancer patients received a median of 14.8 cycles of treatment. Therefore, antitumor activity was observed in patients with *PIK3CA* mutant breast cancer (3 partial response and 3 stable disease of a total of 6 patients) (**Table 2**, **Figure 1**).

**Table 2:** Clinical summary of patients.

	Dose level	Age	Sex	Diagnosis	Site of Metastatic Disease	Prior Systemic Therapies	*PIK3CA* mutation status	Other pathogenic variants	Best response	PFS (months)	Survival
S0001 S0002 S0003 S0004 S0005 S0006 S0007 S0008 S0009 S0010 S0011 S0012	0 0 0 1 1 1 1 1 1 2 2 2	49 50 53 55 61 69 33 59 50 63 40 62	F F F M F F F F F M F F	Breast ca Breast ca CRC CRC Breast ca CRC Breast ca Breast ca CRC CRC Breast ca CRC	Bone, peritoneum Skin, pleura, LNs Lung, liver, peritoneum, LNs Bone, adrenal, LNs Bone Bone Lung, liver, peritoneum, bone Bone, liver Liver Lung, liver Lung Lung, liver, pleura	2 3 2 2 3 2 5 13 4 2 2 2	H1047L E545K E545K - E545K, E726K - E545K H1047R G118D - N345K E542K	*PTEN PTCH1 CDH1* ***KRAS*** *APC TP53 SMAD4* *CDH1 AR* ***KRAS*** ***KRAS*** *TP53 PTEN* *TP53* *TP53* ***KRAS*** *APC*	PR PR PD PD SD PD SD SD PD PD PR PD	26.5 3.5 1.1 1.4 19.6 0.9 2.8 2.5 0.9 1.4 7.5 2.6	Survival Death Death Death Survival Death Death Death Death Death Survival Death

Data are frequency (percentage); ca, cancer; CRC, colorectal cancer; LNs, lymph nodes; NGS, next generation sequencing; PFS, progression-free survival; PR, partial response; SD, stable disease; PD, progression disease.

### 3.4 Safety

Treatment-related AEs occurred in 100.0% (any grade) and 33.3% (Grade 3) of patients. No Grade 4–5 treatment-related AEs occurred. The most frequently reported all-grade, non-hematologic AEs included Gr 2–3 nausea (75.0%), Gr 1–2 diarrhea (50.0%), Gr 1–2 hand-foot syndrome (41.7%), Gr 1–2 anorexia (41.7%), Gr 2 mucositis (33.3%). The most common all-grade, non-hematologic AEs were Gr 1–3 hyperglycemia (75.0%, 9 of 12 patients), and sequentially Gr 1–3 hyperbilirubinemia (50.0%), Gr 1–2 anemia (33.3%).

The Gr 3 treatment-related AEs were nausea (33.3%, 4 of 12 patients), hyperglycemia (16.7%, 2 of 12 patients), hyperbilirubinemia (16.7%), weakness, and gallbladder perforation (all 8.3%). Remarkably, hyperglycemia was dominantly observed in patients with colorectal cancer, with Gr 3 hyperglycemia reported in all 2 colorectal cancer patients. All-grade hyperglycemia was present in all colorectal cancer patients and in 50% of breast cancer patients (**Table 3**).

**Table 3:** Treatment-related adverse events.

	Dose level 0 (n=3)		Dose level 1 (n=6)		Dose level 2 (n=3)		Total (n=12)	
Adverse event	All Gr	Gr 3–5	All Gr	Gr 3–5	All Gr	Gr 3–5	All Gr	Gr 3–5
Dermatologic								
Hand-foot SD	1	0	2	0	2	0	5	0
Skin rash	1	0	0	0	0	0	1	0
Constitutional								
Fatigue	1	0	1	0	0	0	2	0
Weakness	0	0	1	1	1	0	2	1
Weight loss	0	0	0	0	1	0	1	0
Gastrointestinal								
Diarrhea	1	0	4	0	1	0	6	0
Anorexia	1	0	2	0	2	0	5	0
**Nausea**	**2**	**0**	**4**	**2**	**3**	**2**	**9**	**4**
GB perforation	1	1	0	0	0	0	1	1
Pulmonary								
Pleural effusion	1	0	0	0	0	0	1	0
Pain	1	0	0	0	0	0	1	0
Neurology								
Sensory neuropathy	1	0	0	0	2	0	3	0
Mucositis	0	0	2	0	2	0	4	0
Infection								
Cystitis	1	0	0	0	0	0	1	0
Oral candidiasis	0	0	0	0	1	0	1	0
Depression	1	0	0	0	0	0	1	0
Hematology								
Anemia	1	0	2	0	1	0	4	0
Thrombocytopenia	0	0	0	0	0	0	0	0
Neutropenia	0	0	2	1	0	0	2	1
Chemistry								
**Hyperglycemia**	**2**	**0**	**4**	**1**	**3**	**1**	**9**	**2**
AST increased	0	0	1	0	0	0	1	0
Hyperbilirubinemia	2	0	2	1	2	1	6	2

Data are frequency; SD, syndrome; GB, gall bladder; AST, Aspartate transaminase.

The bold values are frequent treatment-related adverse events.

### 3.5 Pharmacokinetics

**Figure 2:**
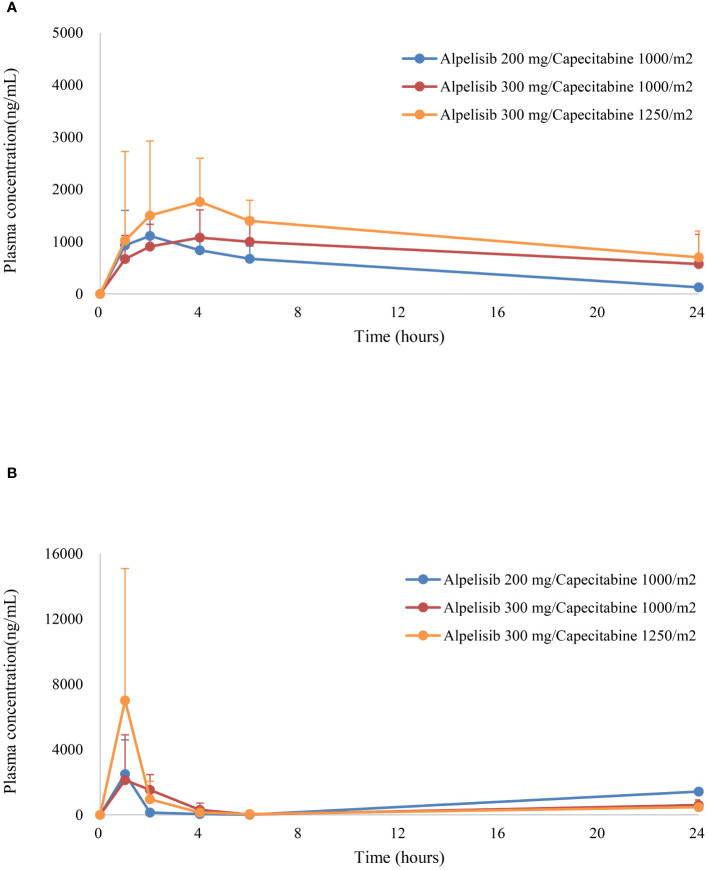
Plasma concentrations (mean ± standard error) of **(A)** alpelisib, and **(B)** capecitabine versus time.

The pharmacokinetics of alpelisib and capecitabine were evaluated in this study (**Figure 2**, **Table 4**). The mean peak plasma concentrations (Cmax) of alpelisib at dose levels 0, 1, and 2 were 1,167.8 ng/mL, 1,235.3 ng/mL, and 2,035.6 ng/mL, respectively, with a time to reach peak concentration (tmax) of 2–4 hours. The area under the concentration-time-curve (AUC) of alpelisib increased with dose escalation, ranging from 4,930.5 ng·h/mL to 8,192.2 ng·h/mL. The half-life of alpelisib was longer at dose level 1 (9.29 hours) compared to dose level 0 (6.5 hours) and dose level 2 (4.3 hours).

**Table 4:** Pharmacokinetics parameters of alpelisib and capecitabine.

	Dose level 0 Alpelisib 200mg+ Capeciabine 1000mg/m^2^ (n=3)	Dose level 1 Alpelisib 300mg+ Capeciabine 1000mg/m^2^ (n=6)	Dose level 2 Alpelisib 300mg+ Capeciabine 1250mg/m^2^ (n=3)
Alpelisib			
c_max_ t_max_	1167.8 (844.7–1699.7) 2 ± 0.6	1235.3 (705.6–1963.3) 4 ± 1.8	2035.6 (942.4–3567.1) 4 ± 2.1
AUC_0-6h_	4930.5 (3201.5–6991.2)	5068.9 (2006.4–8978.1)	8192.2 (2924.5–15893.9)
t_1/2_	6.5 (3.65–10.9)	9.29 (4.1–14.5)	4.3 (4.3-NC)
Capecitabine			
c_max_ t_max_	2498.0 (1135.0–4907.7) 1.0 ± 0.0	4159.0 (1925.9–7980.2) 2.0 ± 1.9	7004.5 (746.0–18227.9) 1 ± 0.0
AUC_0-6h_	2674.4 (1175.2–5310.2)	5995.5 (3471.4–10874.7)	8710.8 (858.2–22483.8)
t_1/2_	0.89 (0.89-NC)	0.63 (0.44–0.89)	0.73 (0.39–1.31)

Data are mean (range) and SD. cmax, peak plasma concentration; t_max_, time to reach peak plasma concentration; AUC, area under the concentration-time-curve; t_1/2_, half-life of time; NC: Not calculated. AUC: ng·h/mL, Cmax: ng/mL, t_max_ & t_1/2_: hour.

For capecitabine, the mean Cmax at dose levels 0, 1, and 2 were 2,498.0 ng/mL, 4,159.0 ng/mL, and 7,004.5 ng/mL, respectively, with a tmax of 1–2 hours. The AUC of capecitabine increased with dose escalation, ranging from 2,674.4 ng·h/mL to 8,710.8 ng·h/mL. The half-life of capecitabine was shortest at dose level 1 (0.63 hours) and longest at dose level 2 (0.73 hours).

Overall, the pharmacokinetics of alpelisib and capecitabine were consistent with previous studies. No pharmacokinetic interactions were observed between the two drugs. However, given the limited sample size and the need for more comprehensive pharmacokinetic analyses, further studies with larger cohorts are needed to provide a better understanding of the effects of dose expansion of the combination therapy.

## 4 Discussion

This phase Ib study explored the safety, MTD, and PR2D of the combination of capecitabine with the oral *PIK3CA* inhibitor alpelisib in patients with advanced solid tumors refractory to standard therapy. The results showed that the combination treatment was generally well-tolerated and had an acceptable toxicity profile. Only one DLT, which was grade 3 hyperglycemia, was observed in one out of six patients treated at dose level 1. Based on our study’s definition, we identified the MTD and RP2D as alpelisib 300 mg daily continuously combined with 1,250 mg/m^2^ capecitabine twice daily for 14 days in 3-weekly cycles. Therefore, we planned further phase II trials to evaluate the efficacy and safety of this combination therapy using the established dosing regimen.

Hyperglycemia was the most commonly reported treatment-related adverse event and the only dose-limiting toxicity observed in our study. Hyperglycemia is a well-known side effect of PI3K-AKT-mTOR pathway inhibitors. The inhibition of this pathway can lead to abrogated insulin function, impaired insulin secretion, and the development of insulin resistance, resulting in hyperglycemia (2, 4, 31). Previous studies have reported the incidence of hyperglycemia associated with PI3K-AKT-mTOR pathway inhibitors in large phase III clinical trials ranging from 13 to 63.7% and high-grade hyperglycemia from 4 to 32.7% (32–34). The incidence of hyperglycemia may also differ depending on the combination drug, disease stage, and alpelisib dose used. For example, in the phase 1b study of triple-combination therapies (encorafenib and cetuximab with alpelisib) in colorectal cancer (*BRAF*-mutant), the incidence of hyperglycemia was 39.3% (16), while a phase II study of alpelisib plus fulvestrant in hormone receptor-positive breast cancer resulted in 29% of hyperglycemia (35). In a phase III study (SOLAR-1), hormone receptor-positive breast cancer showed a higher incidence of hyperglycemia at 63.7% (12). While the frequency of hyperglycemia by cancer type is not well-known in previous clinical studies, it is clear that the severity and incidence of hyperglycemia can vary based on several factors.

In our study, all-grade hyperglycemia was reported in 66.7% (2/3) of patients in the alpelisib 100 mg BID group (dose level 0) and 77.8% (7/9) of patients in the alpelisib 150 mg BID group (dose level 1,2). Grade 3 hyperglycemia was observed in 16.7% (2/12) of patients. The median time to onset of all-grade hyperglycemia was 3.1 weeks (range, 1.0 to 8.7). Although the number of patients was small, there appeared to be a difference in hyperglycemia incidence according to cancer type. Specifically, hyperglycemia was reported in all colon cancer patients and in half of the breast cancer patients. In our study, colon cancer patients appeared to be at a higher risk of hyperglycemia, likely due to the presence of several risk factors such as old age, history of bowel resection, and use of medication that can increase the risk of hyperglycemia. The median age of colon cancer patients was higher than that of breast cancer patients (58.5 years vs. 49.5 years), and all colon cancer patients underwent partial colorectal resection. Furthermore, most colorectal cancer patients were enrolled as third-line chemotherapy. They received FOLFOX (folinic acid, fluorouracil, and oxaliplatin) and FOLFIRI (folinic acid, fluorouracil, and irinotecan)-based chemotherapy in the previous first and second-line treatments, which may have made them more vulnerable to hyperglycemia due to long exposure to dexamethasone with standard pre-medication. Therefore, our findings suggest that clinicians should exercise caution when administering alpelisib and capecitabine to colorectal cancer patients with risk factors for hyperglycemia and closely monitor blood glucose levels during treatment. Further studies are needed to evaluate the safety of this combination therapy using the established dosing regimen.

Other adverse events of combination therapy were nausea, stomatitis, and hand-foot syndrome. The hand-foot syndrome can be attributed to capecitabine since this has not been observed before in single-agent alpelisib trials. This well-known side effect of capecitabine resulted in dose reductions of capecitabine in our patients. As previously known in the study, the mean time until all grades of hand-foot syndrome occurred was 6.1 weeks (range, 3.1 to 15.0), as was previously known from the study. Although stomatitis is a common adverse event of both capecitabine and alpelisib as a single agent, this overlapping toxicity remained mild to moderate in severity in this study and was not dose-limiting.

The combination of alpelisib and capecitabine demonstrated sustained clinical activity in some breast cancer patients with *PIK3CA* mutations. Two patients with breast cancer are still on the study for over 12 months Median progression-free survival (PFS) of 10.4 months showed promising results in patients with heavily-treated breast cancer. In particular, three patients had more than 10 cycles of treatment and had the following mutations: *PIK3CA* H1047L, *PTEN* 317_318del, *PTCH1* D898N, *CDH1* S649fs; *PIK3CA* E545K, *PIK3CA* E726K, *CDH1* R598*, *AR* P549S; and *PIK3CA* N345K TP53 M237I, respectively, which PIK3CA mutation points were heterogeneous. Although this studies had small sample size, previous studies have shown that *CDH1* mutation might enhance the effectiveness of *PIK3CA* inhibitors when present together (36, 37).

This study did not show significant clinical benefits in colorectal cancer patients compared to breast cancer. This suggests that the role of *PIK3CA* mutation as a driver mutation may vary depending on the cancer type. The median PFS for this treatment was only 1.4 months. This could be due to the absence of *PIK3CA* mutation in half of colorectal cancer patients, and the frequent co-occurrence of *KRAS* pathogenic variants with *PIK3CA* mutations, leading to a shorter reported PFS and potentially reduced activity against *PIK3CA* inhibitors. Studies have shown that tumors with aberrant activation of the RAS/RAF/MEK/ERK pathway, such as *KRAS*-mutant cancers, do not respond to PI3K pathway inhibitors (38–40). One study of pictilisib (GDC-0941) found that patients with both *PIK3CA* and *KRAS* mutations had a lower response rate and shorter progression-free survival compared to those with only a *PIK3CA* mutation (41). However, further research is needed to confirm and expand upon these findings.

The results of our study demonstrate that the pharmacokinetics of alpelisib and capecitabine are not affected when these agents are co-administered in patients with advanced cancer. In particular, the Cmax and AUC values of both drugs were similar across all dose levels, indicating that there were no pharmacokinetic interactions between alpelisib and capecitabine. Our study also found that the time to reach peak plasma concentration (tmax) was consistent with previous reports of alpelisib and capecitabine. These findings suggest that it is safe to combine these two agents in patients with advanced cancer. Overall, the pharmacokinetic results of our study support the safety and tolerability of the alpelisib-capecitabine combination and provide a basis for further investigation of this combination in larger phase II clinical trials. Additional pharmacokinetic analyses may be useful in these trials to further characterize the pharmacokinetics of alpelisib and capecitabine and to optimize dosing strategies for this combination therapy.

Limitations of this study include the small number of patients, no control groups, and data for a single institution. In addition, since our study only enrolled breast and colorectal cancer patients, our findings may not be generalizable to all solid tumors, and further studies are needed for other types of cancer. Although our study has reached several endpoints, definitive conclusions cannot be drawn with respect to the synergistic effects of this combination. Moreover, we currently lack clear biomarkers to predict which patients have long-term responses. In addition, insufficient pharmacokinetics and pharmacodynamic analyses are requested better to understand the effects of dose reduction and discontinuation.

In conclusion, our study demonstrated that the combination of alpelisib 300 mg daily and capecitabine 1,250 mg/m^2^ twice daily for 14 days in 3-weekly cycles is a safe and well-tolerated treatment option for patients with advanced cancers harboring *PIK3CA* mutations. Our findings also indicate that this combination therapy achieved prolonged clinical benefits in a significant number of patients. Notably, toxicities associated with this treatment were generally manageable, and no unexplained severe toxicities were reported.

The current therapies for *PIK3CA* mutant advanced solid tumors are being explored in variable drug combinations. A larger prospective study and following experiment should be conducted to elucidate the role of *PIK3CA* mutations in solid tumors. Overall, our study suggests that the combination of alpelisib and capecitabine could be a promising treatment option for patients with *PIK3CA* mutant advanced cancers. Larger studies to validate our results and further investigation is needed in phase II trial.

## Data availability statement

The datasets presented in this study can be found in online repositories. The names of the repository/repositories and accession number(s) can be found in the article/**Supplementary Material**.

## Ethics statement

The studies involving humans were approved by the institutional review boards of Korea University Anam Hospital (2020AN0539) and by the FDA. The studies were conducted in accordance with the local legislation and institutional requirements. The participants provided their written informed consent to participate in this study.

## Author contributions

AL: Data curation, Formal analysis, Writing – original draft, Writing – review & editing, Conceptualization, Methodology, Visualization. BK: Formal analysis, Writing – review & editing. JK: Data curation, Writing – review & editing. MH: Data curation, Writing – review & editing. KP: Conceptualization, Writing – review & editing. YK: Conceptualization, Writing – review & editing. SL: Conceptualization, Data curation, Supervision, Writing – original draft, Writing – review & editing.
