# Supplementary material for: Phase Ib and pharmacokinetics study of alpelisib, a PIK3CA inhibitor, and capecitabine in patients with advanced solid tumors
Source: Front Oncol. 2024 Jul 12;14:1390452. doi: 10.3389/fonc.2024.1390452 (PMC11272611; doi:10.3389/fonc.2024.1390452)
Supplement: Supplementary file 1 [file DataSheet_1.docx]

**Supplement TABLE 1.** Inclusion, exclusion criteria

Inclusion criteria

1. Subject has signed the informed consent form (ICF) prior to any screening procedures being performed

2. Age ≥ 19 years old of male and female

3. At each phase of the trial, subjects who meet the following requirements in each phase will be enrolled.

- Phase Ib: subjects with a histologically-confirmed, advanced/recurrent solid tumor who have progressed on standard therapy or whose disease does not have established standard therapy and not limited to PIK3CA mutation.

- Phase II: subjects with histologically confirmed, PIK3CA mutated, metastatic colorectal cancer that have progressed after treatment with two prior standard chemotherapeutic agents with targeted agents such as cetuximab, bevacizumab, aflibercept (Tissue samples of colorectal cancer patients must contain just PIK3CA gene alterations. e.g. single nucleotide variants, small indels, amplifications, structural variation etc. using targeted panel sequencing.)

(If the subject received adjuvant chemotherapy after curative surgery and lymph node dissection for colorectal cancer, the adjuvant chemotherapy is considered to be the first-line palliative chemotherapy if the disease recurred during adjuvant chemotherapy or within 6 months after the completion of adjuvant chemotherapy.)

4. Patient has evaluable disease as per RECIST 1.1. (Measurable lesions are not mandatory for study inclusion.)

5. ECOG performance status 0-1

6. Patient has adequate bone marrow and organ function as defined by the following laboratory values:

- absolute neutrophil count (ANC) ≥ 1.5 x 109/L

- hemoglobin ≥ 9.0 g/dL

- platelet ≥ 100 x 109/L

- serum creatinine ≤ ULN (upper limit of normal) or serum creatinine clearance > 50 mL/min

- total bilirubin: ≤ 1.5 × ULN

subjects with a bile duct obstruction will be eligible if they meet the criteria after appropriate bile drainage; Patients with Gilbert syndrome should also be included after confirming that the total bilirubin level is ≤ 1.5 x ULN in a follow-up screening test.

- INR ≤1.5

- potassium within normal limits, or corrected with supplements

- fasting Serum amylase ≤ 2 × ULN

- fasting Serum lipase ≤ ULN

- Phase Ib: alanine aminotransferase (AST) and aspartate aminotransferase (ALT) ≤ 3 x ULN (regardless of liver metastases)

- Phase II: AST and ALT ≤ 3 x ULN if liver metastases are absent, or AST and ALT ≤ 5 x ULN if liver metastases are present.

7. Adequate cardiac function: QTc ≤480 msec; if QTc exceeds 480 msec, subjects can be enrolled if the average QTc value is less than 480 msec by measuring 3 times consecutively in total.

8. The subject is able to swallow and retain oral medication

9. Serum β-HCG test negative within 14 days before the first administration of the study treatment (women of childbearing potential only).

10. Requirement for contraception must be observed by the subject.

- women of child-bearing potential (All women who have physiological potential to be pregnant unless adequate contraception is used during the study treatment and the 4 weeks following the discontinuation of the study treatment); subjects can be enrolled if they use the following effective contraceptive methods:

- subjects must abstain from any form of sexual intercourse if the complete abstinence is preferred and consistent with their daily life style. Periodic abstinence (e.g., calendar rhythm method, symptom-thermal method, etc.) and external ejaculation methods are not acceptable contraceptive methods.

- sterilization procedure for women: surgical bilateral oophorectomy with or without hysterectomy; tubal ligation at least 6 weeks prior to enrollment into this study. Bilateral oophorectomy alone is allowed if the subject’s reproductive status is confirmed through a follow-up evaluation of hormone levels.

- vasectomized partner (at least 6 months prior to screening). For a woman participating in clinical trials, the male partners who underwent vasectomy must be the only partner during this study.

- oral contraceptives that are prone to drug-drug interactions may not be effective due to a potential CYP3A4 interaction with alpelisib. Contraception must therefore include a condom and one of followings.

a. medroxyprogesterone injections (e.g., Depo-provera)

b. use of intrauterine device (IUD) or Intrauterine system (IUS, e.g. levonorgesterol intrauterine system; e.g., Mirena)

c. blocking contraceptive method: Condom or blocking cap with spermicide foam/gel/film/cream/ vaginal suppository.

- with appropriate clinical profiles (e.g., appropriate age and history of vasomotor symptoms), if the subject was amenorrhea for more than 12 months or received surgical bilateral oophorectomy (with or without hysterectomy) or tubal ligation at least 6 weeks prior to the screening period, the subject is considered to be postmenopausal and not considered to have childbearing potential. In the case of oophorectomy alone, the reproductive status of the woman must be confirmed by a follow-up hormone level assessment.

- men must use condoms in sexual relations during study medication and after discontinuation of treatment (one month after last dose).

Exclusion criteria

1. Patient has received previous treatment with a PI3K or AKT inhibitor. (Note prior mTOR inhibitor treatment is allowed.)

2. Patient has received previous capecitabine in metastatic setting.

3. Patient has a known or suspicious hypersensitivity to capecitabine or other products containing fluorouracil.

4. Any cytotoxic chemotherapy from a previous treatment regimen within 14 days. If the subject received an investigational drug from another clinical trial, the subject can be enrolled after 2 weeks of last administration and more than 5 x half–life of the investigational drug. If monoclonal antibody therapy was given, the subject can be enrolled after four weeks after the last does.

5. Active central nervous system (CNS) lesions (i.e., those with radiologically unstable or symptomatic brain lesions). For those who receive radiation or surgical treatment, the subject can be enrolled if the subject is maintained without steroid therapy and the evidence of CNS disease progression for more than 4 weeks. However, patients with leptomeningeal metastases are excluded.

6. Patient has currently documented pneumonitis/interstitial lung disease

7. Patient has not recovered to ≤ grade 1 (except alopecia) from related adverse effects of any prior anticancer therapy

8. Radiotherapy with a wide field (more than 30% of the bone marrow) of radiation within 4 weeks or radiotherapy with a limited field of radiation for palliation within 2 weeks of the first dose of study treatment.

9. Patient who has undergone major surgery ≤ 4 weeks prior to starting study treatment or who has not recovered from adverse effects of such procedure.

10. Patient has a clinically significant cardiac disease or impaired cardiac function, such as:

- acute coronary syndrome within the 6 months prior to the initiation of study drug (including myocardial infarction or unstable angina, Coronary Artery Bypass Graft surgery, percutaneous coronary intervention and stenting)

- heart failure ≥ grade 2 by New York Heart Association (NYHA) functional classification or that requires treatment

- ejection fraction (EF) <50% on multi-gated acquisition (MUGA) scan or echocardiography examination. MUGA scan or echocardiography is not required as a screening test if there is no current suspicious symptom and past history of heart failure.

- persistent uncontrolled hypertension as defined by: systolic >160 mmHg or diastolic >100 mmHg with or without anti-hypertensive medication. Initiation or adjustment of antihypertensive medication(s) is allowed prior to screening.

- current or past history of clinically significant cardiac arrhythmia, atrial fibrillation, and/or conduction abnormality (e.g. congenital long QT syndrome, complete AV block)

- on screening, inability to determine the QTcF interval on the ECG (i.e.: unreadable or not interpretable) or corrected QT (QTcF) >450 msec for males and >460 msec for females (using Fridericia’s correction). All as determined by screening ECG (mean of triplicate ECGs).

- any risk factors that prolong QTc or increase the probability of arrhythmia, including medication (e.g. heart failure, hypokalemia, congenital long QT syndrome, history of Torsades de Pointes)

11. If the subject was diagnosed with diabetes (irrespective of treatment or symptom) or if the subject has impaired glucose tolerance (with blood glucose of 140-199 mg/dL after 2 hour oral glucose tolerance test (75g)), previous history of gestational diabetes, or steroid-induced diabetes.

12. Patients with impaired gastrointestinal (GI) function or GI disease that may significantly alter the absorption of oral BYL719 (e.g. untreated peptic ulcer disease, uncontrolled nausea, vomiting, diarrhea, malabsorption syndrome, or wide small bowel resection).

13. History of acute pancreatitis within 1 year of screening or past medical history of chronic pancreatitis

14. Patient has a known positive serology for human immunodeficiency virus (HIV), active Hepatitis B, and/or active Hepatitis C infection. Hepatitis B carriers may be enrolled if prophylactic use of an antiviral agent with minimal interaction with CYP3A4 is administered to inhibit HBV activation (e.g., entecavir, adefovir)

15. Concomitant medication of strong or moderate inducers or inhibitors of CYP3A4 before the first dose of study treatment (In this case, if the drug is stopped for more than 1 week and changed to another drug that does not affect CYP3A4, then the subject can be enrolled.)

16. Inhibitors of BCRP

17. Subjects with unresolved osteonecrosis of the jaw

18. Subject has a history of severe cutaneous reactions like Stevens-Johnson-Syndrome (SJS), Erythema Multiforme (EM), Toxic Epidermal Necrolysis (TEN), or Drug Reaction with Eosinophilia and Systemic Symptoms (DRESS).

19. History of other primary cancer. Exceptions are as follows:

- adequately treated non-melanoma skin cancer (basal cell or squamous cell carcinoma), curatively treated in situ cancer of the cervix or stage I bladder cancer, completely resected thyroid cancer without distant metastasis in which all treatment has been completed (Appropriate wound healing is required prior to clinical trial enrollment)

20. History of allogeneic bone marrow transplantation or organ transplantation

21. As judged by the Investigator, all other symptoms and associated disease for which the investigator determined that participation in this study is contraindicated (e.g. Infection/inflammation; severe liver dysfunction; bilateral diffuse interstitial lung disease; uncontrolled renal disease; unstable heart and lung disease; hemorrhagic disease; intestinal obstruction; unable to swallow oral pills; social and psychological problems, etc.)

22. Medical, psychiatric, cognitive, or other conditions that may interfere with the ability of the subject to understand the subject information, provide the informed consent, follow the protocol process, or complete the clinical trial

**Supplement TABLE 2.** Definition of Dose Level

**Dose level -1 (1 cycle = 3 weeks)**

| Study treatment | Route of administration | Dose | Frequency |
| --- | --- | --- | --- |
| Alpelisib | Oral | 100mg | BID every day |
| Capecitabine | Oral | 850mg/m^2^ | BID D1-D14 on  D15-D21 off |

**Dose level 0 (1 cycle = 3 weeks)**

| Study treatment | Route of administration | Dose | Frequency |
| --- | --- | --- | --- |
| Alpelisib | Oral | 100mg | BID every day |
| Capecitabine | Oral | 1000mg/m^2^ | BID D1-D14 on  D15-D21 off |

**Dose level 1 (1 cycle = 3 weeks)**

| Study treatment | Route of administration | Dose | Frequency |
| --- | --- | --- | --- |
| Alpelisib | Oral | 150mg | BID every day |
| Capecitabine | Oral | 1000mg/m^2^ | BID D1-D14 on  D15-D21 off |

**Dose level 2 (1 cycle = 3 weeks)**

| Study treatment | Route of administration | Dose | Frequency |
| --- | --- | --- | --- |
| Alpelisib | Oral | 150mg | BID every day |
| Capecitabine | Oral | 1250mg/m^2^ | BID D1-D14 on  D15-D21 off |

• Three subjects will be enrolled at each dose level.

• If no DLT occurred for one cycle (21 days) at each level, three study subjects will be enrolled at the next dose level.

• If DLT occurs in more than 2 out of 3 people at each level, 3 more people will be enrolled in a lower dose level.

• If DLT occurs in 1 out of 3 subjects at each level, additional 3 subjects will be enrolled in the same dose level. If DLT occurs in 1 out 6 subjects, three study subjects will be enrolled at the next dose level. If DLT occurs in more ≥2 out of 6 subjects, three study subjects will be enrolled at the previous (lower) dose level (up to 6 subjects). If 6 subjects are registered at the lower dose level and DLT occurs in less than 2 people, MTD is determined.

• If DLT occurs in ≥2 out of 6 subjects at any level, it is considered as exceeding the MTD, and the previous dose level is defined as MTD.

The recommended phase II dose (RP2D) is determined according to the MTD determined in Phase 1b part. Generally, the MTD is determined as RP2D, but the RP2D may be determined at lower doses than the MTD, considering both the profiles of all adverse events and the patients’ tolerability.

• Initially, dose level 1 will be assessed. If DLT occurs in 0 of 3 or ≤1 of 6 patients, dose level 2 will be assessed in the following order. If dose level 1 is estimated to exceed MTD, then dose level -1 is assessed. If DLT occurs in 0 of 3 or ≤1 of 6 patients at dose level -1, the dose level 0 will be assessed. At MTD or RP2D, toxicity should be assessed in at least 6 patients.
